# UPR^mt^ activation improves pathological alterations in cellular models of mitochondrial diseases

**DOI:** 10.1186/s13023-022-02331-8

**Published:** 2022-05-17

**Authors:** Juan M. Suárez-Rivero, Carmen J. Pastor-Maldonado, Suleva Povea-Cabello, Mónica Álvarez-Córdoba, Irene Villalón-García, Marta Talaverón-Rey, Alejandra Suárez-Carrillo, Manuel Munuera-Cabeza, Diana Reche-López, Paula Cilleros-Holgado, Rocío Piñero-Perez, José A. Sánchez-Alcázar

**Affiliations:** 1 Centro Andaluz de Biología del Desarrollo (CABD), Consejo Superior de Investigaciones Científicas, Universidad Pablo de Olavide, Carretera de Utrera Km 1, 41013 Seville, Spain; 2 Centro de Investigación Biomédica en Red: Enfermedades Raras, Instituto de Salud Carlos III, 41013 Seville, Spain

**Keywords:** Mitochondria, UPRmt, Tetracycline, GFM1

## Abstract

**Background:**

Mitochondrial diseases represent one of the most common groups of genetic diseases. With a prevalence greater than 1 in 5000 adults, such diseases still lack effective treatment. Current therapies are purely palliative and, in most cases, insufficient. Novel approaches to compensate and, if possible, revert mitochondrial dysfunction must be developed.

**Results:**

In this study, we tackled the issue using as a model fibroblasts from a patient bearing a mutation in the *GFM1* gene, which is involved in mitochondrial protein synthesis. Mutant *GFM1* fibroblasts could not survive in galactose restrictive medium for more than 3 days, making them the perfect screening platform to test several compounds. Tetracycline enabled mutant *GFM1* fibroblasts survival under nutritional stress. Here we demonstrate that tetracycline upregulates the mitochondrial Unfolded Protein Response (UPR^mt^), a compensatory pathway regulating mitochondrial proteostasis. We additionally report that activation of UPR^mt^ improves mutant *GFM1* cellular bioenergetics and partially restores mitochondrial protein expression.

**Conclusions:**

Overall, we provide compelling evidence to propose the activation of intrinsic cellular compensatory mechanisms as promising therapeutic strategy for mitochondrial diseases.

**Supplementary Information:**

The online version contains supplementary material available at 10.1186/s13023-022-02331-8.

## Background

Mitochondria, the power plant of the cells, are semi-autonomous cellular organelles that are found in virtually all eukaryotic cells [1]. They are the result of a symbiotic relationship between the precursors of eukaryotic cells and α-proteobacteria [2]. According to Lynn Margulis’ Endosymbiotic Theory of Evolution [3], these bacteria were engulfed by eukaryotic cells’ ancestors to eventually develop a mutually beneficial relationship [4]. Most mitochondrial proteins are encoded in nuclear DNA (nDNA), however, mitochondrial DNA (mtDNA)-encoded proteins are necessary to ensure mitochondrial function [5–7]. Apart from ATP synthesis, these organelles are involved in numerous processes such as cell death induction [8], thermogenesis maintenance [9], the regulation of intracellular Ca^2+^ homeostasis [10], redox state control [11] or steroid synthesis [12].

Mitochondrial diseases are a group of hereditary and highly heterogeneous disorders that originate as a consequence of mutations on either mitochondrial or nuclear genes encoding mitochondria-targeted proteins [13]. These mutations disrupt mitochondrial function, resulting in deficient ATP generation and reactive oxygen species (ROS) overproduction. Such energy shortage is considered to be the triggering factor for most mitochondrial pathologies, which end up becoming multisystemic disorders [14]. The prevalence of these disorders is of 1 every 5000 individuals, reason why they are categorized as rare diseases [15]. One of their most characteristic features is the high variability in clinical presentation, being the most common symptoms muscle weakness and exercise intolerance, neurodegeneration, neurosensory hearing loss, axonal neuropathy, gastrointestinal disorders, diabetes mellitus, renal tubular acidosis and hypertrophic cardiomyopathy [16]. Unfortunately, available therapies are merely capable of alleviating the general disease symptomatology [17].

The findings of this work give insight into a new perspective for a therapeutic approach against mitochondrial diseases and explain the mechanisms supporting its efficacy. Fibroblasts derived from a patient (GFM1) bearing two inherited, pathogenic heterozygous mutations on the G elongation factor mitochondrial 1 (*GFM1*) were used as a model of mitochondrial disease [18]. *GFM1*(EF-G1, MIM#606639) is a nuclear gene that encodes one of the three mitochondrial translation elongation factors that enable mitochondrial protein synthesis. Hence, impairment of EF-G1 function severely compromises the elongation process of mitochondrial-encoded proteins, being the electron transport chain disrupted as a consequence.

Tetracyclines are a family of antibiotics that inhibit bacterial protein synthesis [19]. Their extensive application against human and animal infections is explained by their potent antimicrobial activity and safety. Tetracycline also presents a series of properties that may prove of high interest such as antioxidant activity [20], poly (ADP-ribose) polymerase-1 (PARP-1) inhibition [21] and metalloproteases (MMP) inhibition [22]. Tetracycline usage could trigger mild proteotoxic stress on mitochondria, eventually leading to the activation of proteostasis mechanisms such as the mitochondrial unfolded protein response (UPR^mt^). This phenomenon is known as “hormesis”, where low exposition to toxins or stressors promotes the activation of a favorable biological response [23]. Activation of the UPR^mt^ encompasses several mechanisms that are aimed to repair and boost the recovery of accumulated damaged proteins [24]. Given their proteostatic character, these mechanisms could potentially enhance cellular homeostasis on mitochondrial diseases patients [25, 26].

## Results

### Tetracycline treatment promotes survival of mutant *GFM1* fibroblasts in galactose medium

**Fig. 1:**
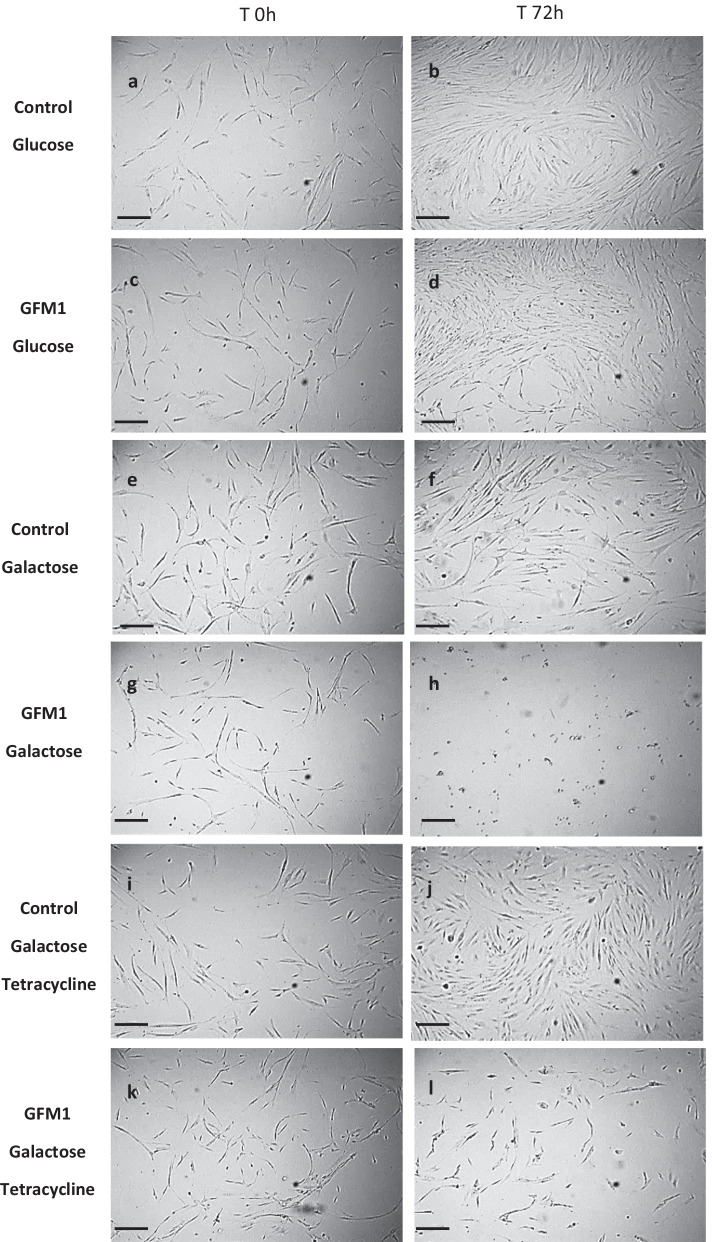
Galactose medium screening and tetracycline treatment. Cells were initially seeded in glucose. After 3 days, glucose medium was changed to galactose. Images were acquired right after changing the medium to galactose (T0) and 72 h later (T72). In optimal conditions both cell lines present a similar proliferation rate (**a**, **b**, **c**, **d**). Control cells do not alter their growth rate (**e**, **f**) but mutant *GFM1* cells undergo cell death after 72 h of culture in galactose medium (**g**, **h**). Tetracycline treatment does not affect control cells (**i**, **j**) but triggers the survival of mutant *GFM1 *cells in galactose medium. The proliferation rate of mutant *GFM1* cells in galactose is slower than in normal conditions (**k**, **l**). Cells were cultured for 3 days in glucose, then the medium was changed to galactose and 10 µM tetracycline treatment was refreshed. Cell viability was assessed by live cell imaging counting and trypan blue 0.2% staining. Cell counting and representative images were acquired using the BioTek™ Cytation™ 1 Cell Imaging Multi-Mode Reader. Refer to Additional file 1: Figure S1 for the quantification of cellular proliferation. Scale bar = 40 μm

Since *GFM1* mutations severely compromised mitochondrial function, we developed a cellular screening technique based on nutritional stress in galactose medium. Control fibroblasts cultured in galactose medium grew normally and presented a conventional morphology (Fig. 1f). However, mutant *GFM1* cells undergo cell death and detach from the surface of the culture flasks 72 h after the application of galactose medium, most likely due to the lack of functional mitochondria (Fig. 1h). Contrary to this, mutant *GFM1* cells cultured on a glucose rich medium grew at a comparable rate to control cells (Fig. 1d). This easy and quick screening would enable us to discern which treatments were successful on improving viability of mutant *GFM1* cells. A first set of antioxidants and mitochondrial function enhancers (Coenzyme Q_10_, Carnitine, Ambroxol, AICAR, Bezafibrate, Curcumin and Lipoic acid) was assessed but none of the compounds succeeded on promoting mutant GFM1 cell survival on galactose medium after 72 h. Next, we opted for a strategy aimed at inducing the phenomenon of “mitohormesis”. This phenomenon is defined as a biological response to mild mitochondrial stressors that favours cellular fitness and survival [27]. Thus, to test whether the induction of mitohormesis might be of benefit for mutant *GFM1* cells, tetracycline treatment was evaluated. Tetracycline antibiotics are well known for their broad spectrum activity, spanning a wide range of Gram-positive and -negative bacteria, spirochetes, obligate intracellular bacteria, as well as protozoan parasites [28]. Tetracyclines preferentially bind to bacterial ribosomes and interact with a highly conserved 16S ribosomal RNA (rRNA) target in the 30S ribosomal subunit, arresting translation by sterically interfering with the docking of aminoacyl-transfer RNA (tRNA) during elongation [29–31]. Given that evolutionarily mitochondria and bacteria have a shared origin, tetracycline was highly likely to exert stress on mitochondria and promote UPR^mt^ activation [3].

As expected, almost no differences could be observed on the growth of treated control cells after the switch to galactose medium (Fig. 1j). Nevertheless, 10 μM tetracycline treatment enabled the survival of mutant *GFM1* cells on galactose medium (Fig. 1l), even though their growth rate was slower than in glucose-rich medium (Additional file 1: Figure S1). The ability of other non-tetracycline antibiotics (i.e., ampicillin) to promote cell survival was also evaluated but these were all unsuccessful. Following this finding the efficacy of other members of the tetracycline family, such as doxycycline or minocycline was assessed. Tetracycline-related antibiotics promoted cell survival on galactose medium to different extent depending on the dose applied (Additional file 1: Figure S2).

### Tetracycline treatment increases mitochondrial protein expression of mutant *GFM1 *fibroblasts

**Fig. 2:**
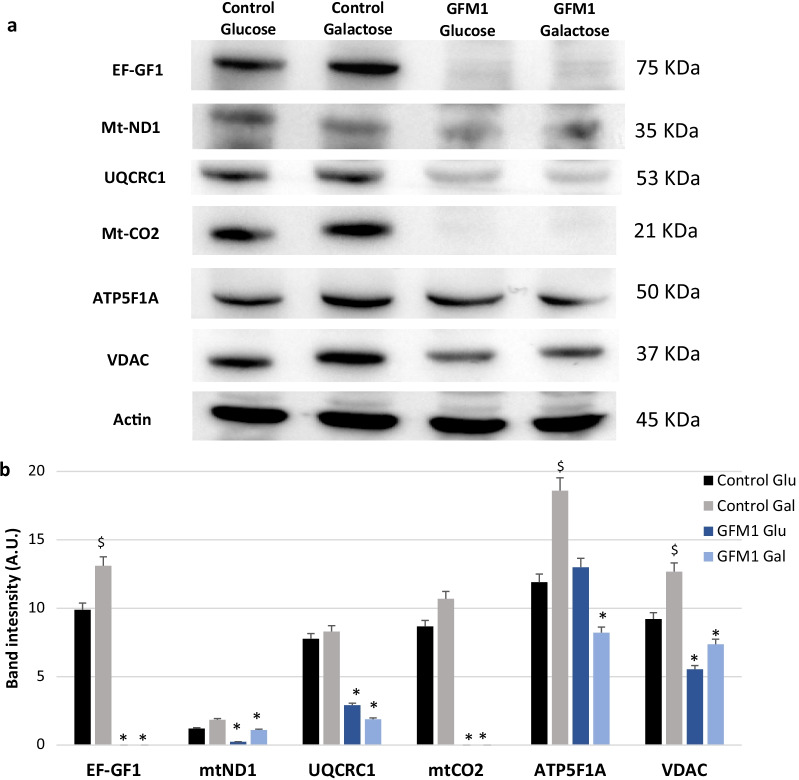
Initial protein expression characterization of mutant *GFM1* cells. **a** Western blot analysis of the mutant protein (EF-G1) and several nuclear (UQCRC1, ATP5F1A) and mitochondrial-encoded (mt-ND1, mt-CO2) proteins forming mitochondrial complexes. VDAC was used as mitochondrial mass marker. Galactose samples were taken after 36 h of medium exposition. A representative actin band is shown, although loading control was additionally checked for every Western blot. **b** Band densitometry of the western blot. Data represents the mean ± SD of 3 independent experiments. *p < 0.05 between control and mutant *GFM1* cells; ^$^*p* < 0.05 between glucose and galactose medium control cells. KDa=Kilodalton, kDa

The protein expression profile of mutant *GFM1* cells was additionally studied. Since the mutation on the *GFM1* gene is reportedly responsible for the aberrant elongation of mitochondrial-encoded proteins, the presence of the mutant protein (EF-G1) and other proteins of the mitochondrial respiratory complexes was assessed by Western blot and immunofluorescence (Fig. 2, Additional file 1: Figure S3 and S4). The proteins included in the Western blot analysis were: Complex I NADH:ubiquinone oxidoreductase core subunit 1 (mt-ND1); Complex III Ubiquinol-Cytochrome C Reductase Core Protein I (UQCR1), being this protein encoded in the nucleus; Complex IV Cytochrome c oxidase II (mt-CO2); Complex V ATP synthase F1 subunit alpha (ATP5F1A), also encoded in the nucleus and finally, voltage-dependent anion channels (VDAC) as mitochondrial mass control. Results showed that most mitochondrial protein levels are reduced in mutant *GFM1* fibroblasts, VDAC included, suggesting a general decrease in mitochondrial mass.

Next, EF-GF1 and mt-CO2 protein expression levels were evaluated by confocal microscopy using Mitotracker DeepRed FM as a marker of mitochondrial mass. A consistent reduction of protein expression levels in mutant *GFM1* cells with respect to control cells was observed (Additional file 1: Figure S3 and S4). This being a clear indication of the profound mitochondrial dysfunction triggered by the *GFM1* mutation.

**Fig. 3:**
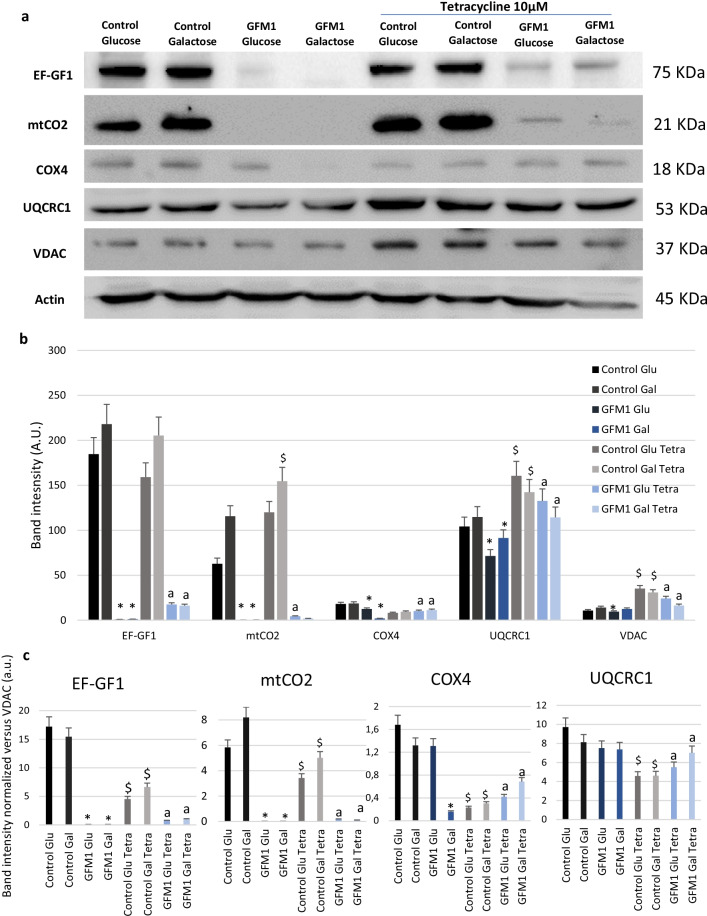
Protein expression levels under tetracycline treatment. **a** Western blot analysis of the mutated protein (EF-G1) and several mitochondrial complex proteins after tetracycline treatment in mutant GFM1 cells. Control and mutant *GFM1* cells were treated with 10 µM tetracycline for 7 days. Cells were seeded in glucose-rich medium. A representative actin band is shown for each assay, although loading control was checked for every Western blot. **b** Band densitometry of the western blot. **c** Normalization of band densitometry versus VDAC. Data represents the mean ± SD of 3 independent experiments. *p < 0.05 between control and mutant *GFM1* cells; ^a^*p* < 0.05 between non-treated mutant *GFM1* and treated mutant GFM1 cells; ^$^*p* < 0.05 between untreated and treated control cells. A.U., arbitrary units. KDa=Kilodalton, kDa

Mitochondrial protein expression levels were then examined after a 7-day under tetracycline treatment (Fig. 3a). Moreover, since Complex IV seemed to be especially compromised by *GFM1* mutation, the expression levels of cytochrome c oxidase subunit 4 (COX4), a nuclear encoded subunit of this complex, was also examined. Tetracycline promoted a significant increase of all mitochondrial proteins’ expression levels. In addition, two other patient cell lines with *GFM1*-related mutations were tested (Additional file 1: Figure S5) and similar results were obtained.

To further understand if the increase of mitochondrial proteins was also associated to a general increase in mitochondrial mass, the band intensity was also normalized to VDAC (Fig. 3c). An increase in mitochondrial mass was indeed observed in *GFM1* fibroblasts after tetracycline treatment.

The increased mitochondrial protein expression levels had a significant impact on cellular homeostasis, as evidenced by the ability of mutant *GFM1* cells to survive in galactose medium. Additionally, a marked disruption of mitochondrial network could be observed in mutant *GFM1 *cells. Interestingly, tetracycline treatment partially recovered mitochondrial network (Additional file 1: Figure S3 and S4).

**Fig. 4:**
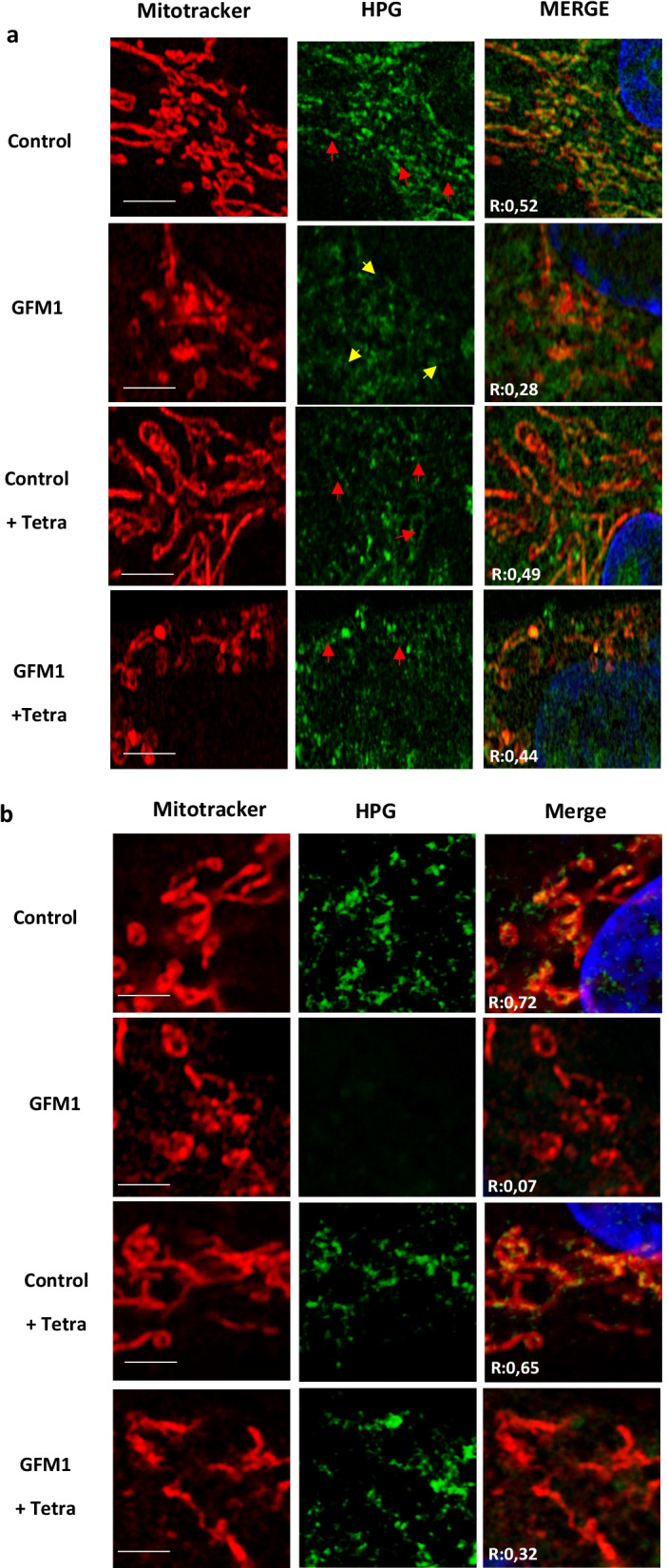
Mitochondrial protein synthesis. **a** Cytosolic and mitochondrial protein synthesis. **b** Mitochondrial protein synthesis. Cycloheximide 50 μg/ml was added for 20 min to inhibit cytosolic protein synthesis previous cell staining. Cells were then treated with 10 μM tetracycline for seven days. Cell staining were performed by incubating with HPG 488 Alexa Fluor and Mitotracker DeepRed FM for 45 min. Then samples were fixed and nuclei stained with Hoescht 1 μg/ml. Red arrows represent positive colocalization with mitochondria and yellow arrows represent negative colocalization. R = Pearson Coefficient of correlation between Mitotracker and HPG. Scale bar in **a** 5 μm; Scale bar in **b** 3 μm. Chloramphenicol and chloramphenicol plus cycloheximide controls are shown in Additional file 1: Figure S6a and b respectively. Three coverslips per condition were analyzed. N = 20 cells. Full colocalization results are shown in Additional file 1: Figure S7

Furthermore, both the total and mitochondrial protein synthesis were monitored with a protocol based on the fluorescent non-canonical amino acid tagging (FUNCAT) procedure [33]. Control cells showed a clear colocalization between the mitochondrial network (Mitotracker) and the protein synthesis reporter (HPG). In contrast, this colocalization was highly reduced in mutant *GFM1* cells and partially restored when mutant cells were treated with tetracycline (Fig. 4 and Additional file 1: Figure S7).

### Tetracycline treatment improves cell bioenergetics of mutant *GFM1* fibroblasts

**Fig. 5:**
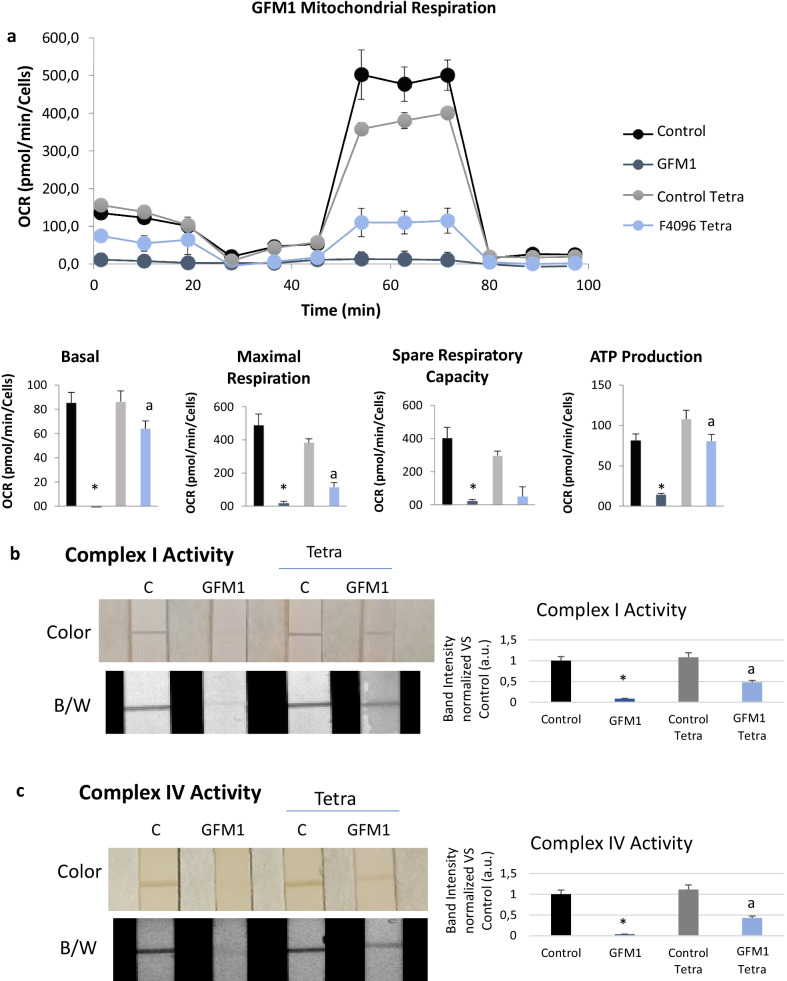
Effect of tetracycline treatment on cell bioenergetics. **a** Mitochondrial respiration profile was measured with a Seahorse XFe24 analyzer. **b** Dipstick results of complex I activity in both colour (bluish-purple) and black/white. **c** Dipstick results of complex IV activity in both colour (yellowish) and black/white. Normalization was performed vs control. Control and mutant *GFM1* fibroblasts were treated with 10 μM tetracycline for 7 days. Cells were seeded at a density of 1.5 × 10^4^ cells/well with 500 µL growth medium (DMEM medium containing 10% FBS and 4,5 g/l glucose). 30 μg of protein were used for each dipstick assay.*p < 0.01 between Control and GFM1 fibroblasts. ^a^p < 0.01 between untreated and treated mutant *GFM1* fibroblasts. OCR, Oxygen Consumption Rate; a.u. (Arbitrary unit)

Having analysed the impact of tetracycline treatment at protein level, we evaluated its ability to improve the bioenergetic activity of mitochondria on mutant *GFM1* cells. For this purpose, a SeaHorse Mitostress assay was performed in control cells, untreated mutant *GFM1* cells and mutant *GFM1* cells treated with tetracycline (Fig. 5a). As expected, untreated mutant *GFM1* fibroblasts presented almost no mitochondrial activity. Promisingly, tetracycline treatment significantly improved mitochondrial basal respiration and ATP production, although the changes in spare respiratory capacity remained non-statistically significant after the treatment. This result proves that mitochondrial proteins’ levels increase under tetracycline treatment and that these proteins are functional and able to partially restore respiration and ATP production.

In addition, the activity of the complexes I (Figs. 5b) and IV (Fig. 5c) was measured by a dipsticks assay. Both in control and mutant *GFM1* cells the activity of such complexes significantly increased after tetracycline treatment with respect to untreated conditions.

### Tetracycline treatment activates UPRmt

Given tetracycline’s ability to enhance protein synthesis, the bioenergetic profile and the viability of mutant *GFM1* cells, our next aim was to elucidate the molecular mechanisms responsible for the efficacy of this antibiotic. It has been recently reported that tetracyclines are activators of the mitochondrial unfolded protein response (UPR^mt^) [34]. The UPR^mt^ comprises diverse, not yet elucidated signaling pathways that control and ensure the maintenance of protein homeostasis within mitochondria [35].

Our hypothesis proposed that tetracycline could act as a mild mitochondrial stressor that promotes the activation of the UPR^mt^ [36]. This effect is known as “mitohormesis” [27] and is regarded as a beneficial mechanism for cellular homeostasis that leads to increased longevity [37] and decreased cancer incidence [38] in several animal models. The UPR^mt^ has been thoroughly studied in *Caenorhabditis elegans* [39–41], however, there is still a considerable lack of information regarding the mechanistic of human UPR^mt^, being the function of several proteins controversial to date. Nonetheless, the activation of UPR^mt^ has already been proposed as a potential treatment for neurodegenerative diseases [42].

#### UPR^mt^ protein profile is increased in tetracycline-treated fibroblasts

To evaluate whether tetracycline treatment promotes UPR^mt^ activation, the expression levels of UPR^mt^-related proteins present in mutant *GFM1* cells exposed to a long-term tetracycline treatment were quantified. An initial tetracycline concentration of 10 µM was applied for 7 days and the dosage was then increased every 7 days (10 µM treatment at day 7, 50 µM treatment at day 14 and 100 µM treatment at day 21). Notably, cell growth and fitness were compromised by the 100 µM treatment, which suggests a toxic effect of tetracycline at high concentrations. The expression levels of the mutant protein, EF-GF1, Eif2α and its phosphorylated and active state (p-Eif2α) were then analysed. The latter is involved in the activation of stress responses, such as the UPR^mt^, since it acts as a sensor of multiple kinds of cellular stress factors [43]. Likewise, the expression levels of ATF4, ATF5 and CHOP, which are canonical human UPR^mt^ activators, were measured [44]. Additionally, we evaluated HSP60 and mtHSP70 which are known chaperones, and SIRT3 to assess the antioxidant mitochondrial axis [45]. Finally, in order to check whether tetracycline treatment promotes mitochondrial biogenesis, PPARgamma coactivator1-alpha (PGC1α), Phospho-PGC1α and Transcription Factor A Mitochondrial (TFAM) expression were examined. These proteins are considered to be canonical activators of mitochondrial biogenesis [46]. Previous studies have reported that UPR^mt^ activation regulates mitochondrial biogenesis or network expansion programming [47, 48].

**Fig. 6:**
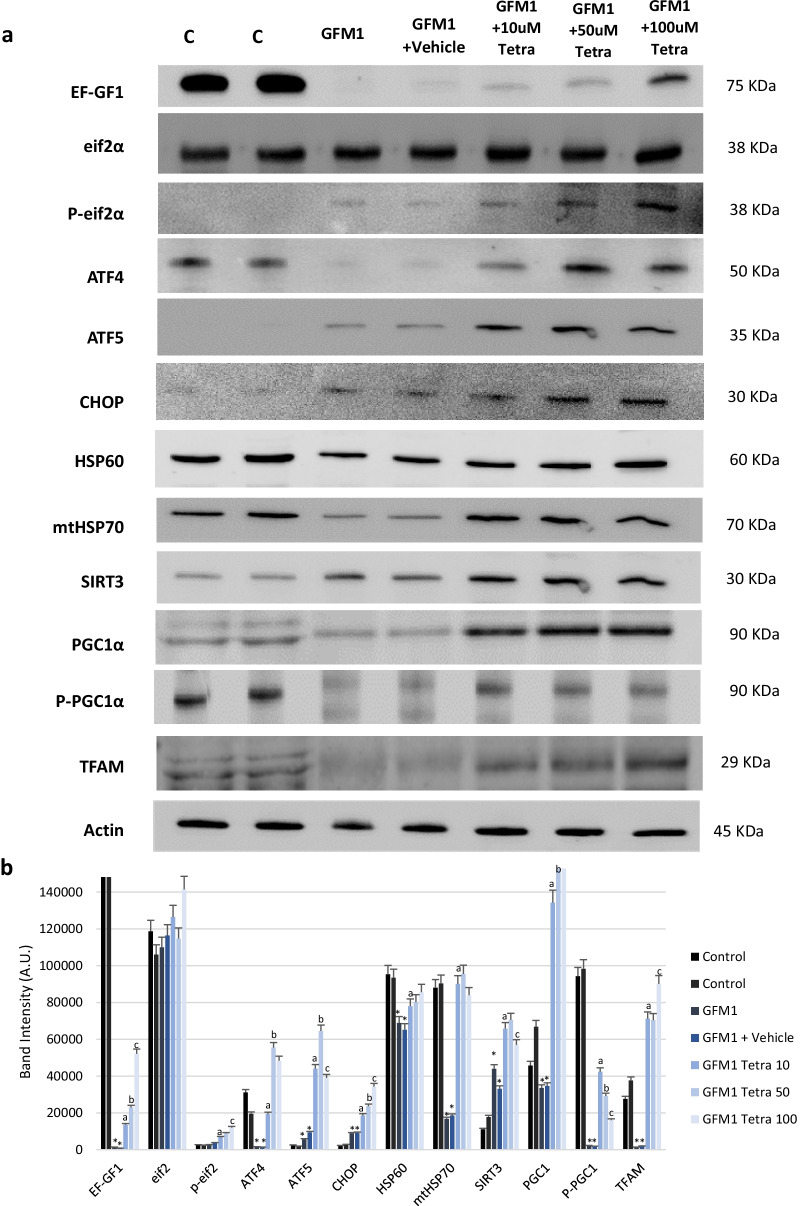
UPR^mt^-related proteins expression under increasing concentrations of tetracycline treatment. **a** Western blot analysis of the mutated protein (EF-G1) and several UPR^mt^-related proteins. Eif2α and P-eif2α are used as Integrated Stress Response (ISR) markers; ATF4, ATF5 and CHOP as canonical UPR^mt^ proteins; HSP60 and mtHSP70 as chaperones; SIRT3 as mitochondrial antioxidant pathway modulator; and PGC1α and TFAM as mitochondrial biogenesis regulators. **b** Band densitometry of the western blot. Data represents the mean ± SD of 3 separate experiments. ^$^p < 0.05 between untreated and treated control cells; *p < 0.05 between control, non-treated and vehicle patients; ^a^*p* < 0.05 Between untreated patient cells and tetracycline (10 μM) treated patient cells; ^b^*p* < 0.05 Between tetracycline (10 μM) treated patient cells and tetracycline (50 μM) treated patient cells; ^c^*p* < 0.05 Between tetracycline (50 μM) treated patient cells and tetracycline (100 μM) treated patient cells. A.U., arbitrary units. Vehicle refers to ethanol, the dissolvent used for tetracycline’s dilution. Cells were sequentially treated with increasing concentrations of tetracycline (10 µM for seven days, then changed to 50 µM up to day 14 and finally changed to 100 µM until day 21). A representative actin band is shown for all assays, although loading control was checked in every Western blot. KDa=Kilodalton, kDa

In these conditions, the increase on the levels of EF-GF1 protein in mutant *GFM1* cells correlated with the increase in dosage and incubation time of tetracycline treatment (Fig. 6). The same trend could be observed for p-Eif2α, ATF5, CHOP and SIRT3, all of which are stress-related proteins. Paradoxically, ATF4 levels were higher in control cells than in mutant *GFM1* cells. This can be explained by the variable functions of this protein in different cell types [49–51]. The same could be stated about the chaperones HSP60 and mtHSP70, which might have a lower expression in mutant *GFM1* cells due to a failure in the compensatory pathways as a consequence of a a prolonged overactivation. After tetracycline treatment, there is an increase in PGC1α, P-PGC1α and TFAM expression levels on *GFM1* cells. Although only TFAM follows a correlation between tetracycline dosage effect and protein expression levels as observed in other proteins.

#### ATF5 knockout promotes cell survival but ATF4 knockout inhibit cell survival of mutant *GFM1* fibroblasts

To further prove UPR^mt^ activation is directly responsible for the improvement of mitochondrial activity in mutant *GFM1* cells, the impact of silencing *ATF5*, a transcription factor regulating the expression of UPR^mt^-related proteins, was assessed on mutant *GFM1* cells. The importance of *ATF5* for the activation of UPR^mt^ has been thoroughly described in the literature [44, 52, 53]. Nonetheless, such importance is in most cases merely assumed since it is a known ortholog of the Activating Transcription Factor associated with Stress 1 (*ATFS-1*) present in *C. elegans.* Still, its precise role on UPR^mt^ initiation is not yet known. In fact, human UPR^mt^ is significantly more complex than *C. elegans’* and some of its features are still not completely understood [54].

**Fig. 7:**
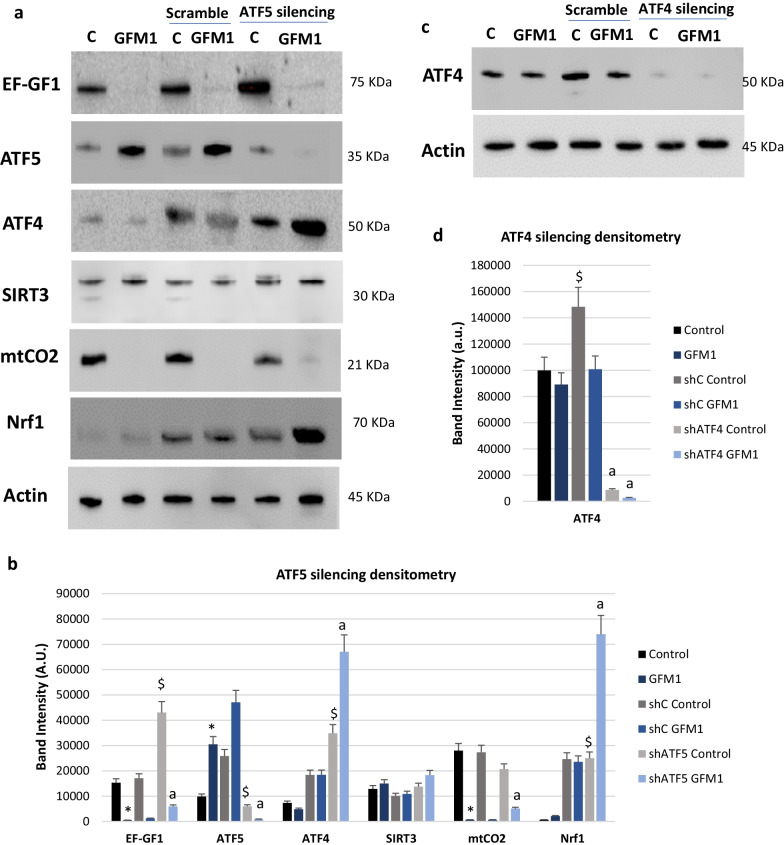
UPR^mt^ proteins after *ATF5* silencing. **a** Western blot analysis of the mutated protein (EF-G1) and several UPR^mt^-related proteins. *ATF5* was silenced using a short hairpin RNA (shRNA) introduced by lentiviral particles against ATF5 RNA plus a puromycin selection marker. ATF5, ATF4, SIRT3 and Nrf1 were selected as UPR^mt^-related proteins, and mtCO2 as mitochondrial marker. **b** Band densitometry of **a**. **c** ATF4 expression levels after *ATF4* silencing. A representative actin band is shown for all assays, although loading control was checked in every Western blot. **d** Band densitometry of Fig. 7c. Data represent the mean ± SD of 3 separate experiments. ^$^*p* < 0.05 between untreated and treated control cells; *p < 0.05 between control and mutant *GFM1* cells; ^a^*p* < 0.05 Between non-silenced and silenced cells; A.U., arbitrary units. Puromycin selection was performed at 2 µg/ml concentration. KDa=Kilodalton, kDa

*ATF5* was silenced with lentiviral particles. Silencing efficacy was tested via Western blot analysis (Fig. 7a, b). Surprisingly, silencing *ATF5 *triggered a mild increase on EF-GF1 and mtCO2 levels, as well as a prominent rise in the amount of other UPR^mt^-related proteins such as ATF4 and Nuclear Respiratory Factor 1 (Nrf1). Given these results, a viability screening assay with shATF5 mutant *GFM1* cells (Additional file 1: Figure S8a) was performed. Interestingly, *ATF5*-silenced mutant *GFM1* cells survived in galactose medium, similarly to tetracycline-treated mutant GFM1 cells. These findings suggest that the loss of *ATF5* also promotes UPR^mt^ activation, presumably by boosting the expression of *ATF4* as a compensatory mechanism. Therefore, *ATF5* silencing would palliate the functional loss of EF-G1.

Additionally, to better understand the link between ATF5 and UPR^mt^ activation we silenced *ATF4* expression, an upstream protein in the UPR^mt^ activation signalling pathway. The importance of this protein for mitochondrial quality control and UPR^mt^ is widely known [55–57]. *ATF4* was also silenced using lentiviruses, following the same strategy that had previously been established to silence *ATF5* (Fig. 7c, d). Surprisingly, upon *ATF4 *silencing mutant *GFM1* cells could not survive, neither on glucose-rich nor galactose media. Moreover, the proliferation of control cells was severely compromised, suggesting the critical relevance of *ATF4* for cell survival (Additional file 1: Figure S8b). In this case, tetracycline treatment did not revert the phenotype originated by the knockdown of *ATF4*.

Due to the prominent death and impaired proliferation of ATF4- deficient cells, the acquisition of samples for Western blot analysis was complicated, being only few samples eventually obtained. Thus, only ATF4 and actin protein levels were measured (Fig. 7c).

### Effect of tetracycline on induced neurons (iNs)

The mutant *GFM1* fibroblasts model provided useful information on the pathophysiology of this disease, however the most affected cell types in the majority of mitochondrial pathologies are muscle cells or neurons [58, 59]. Therefore, direct reprogramming of mitochondrial diseases patient-derived fibroblasts into iNs is an extremely valuable tool to understand the pathogenesis of these disorders. For this reason, control and mutant *GFM1* fibroblasts were direct-reprogrammed to iNs. Reprogrammed cells presented a typical neuron-like morphology and positive immunoreactivity against Tau, a microtubule-associated protein primarily found in neuronal axons of vertebrates' brain. In contrast, unprogrammed cells did not show Tau staining.

Tau+ cells were used to calculate neuronal conversion efficiency (Tau+ cells over the total number of fibroblasts seeded for conversion), which was around 10% in control (9.1 ± 2.1%) and 12% in mutant *GFM1* cells (12.5 ± 3.1%) cells. Neuronal purity (Tau+ cells over the total cells in the plate after reprogramming) was around 20% (18.2 ± 2%) in control cells and up to 22% (20.1 ± 2.6%) in mutant *GFM1* cells.

**Fig. 8:**
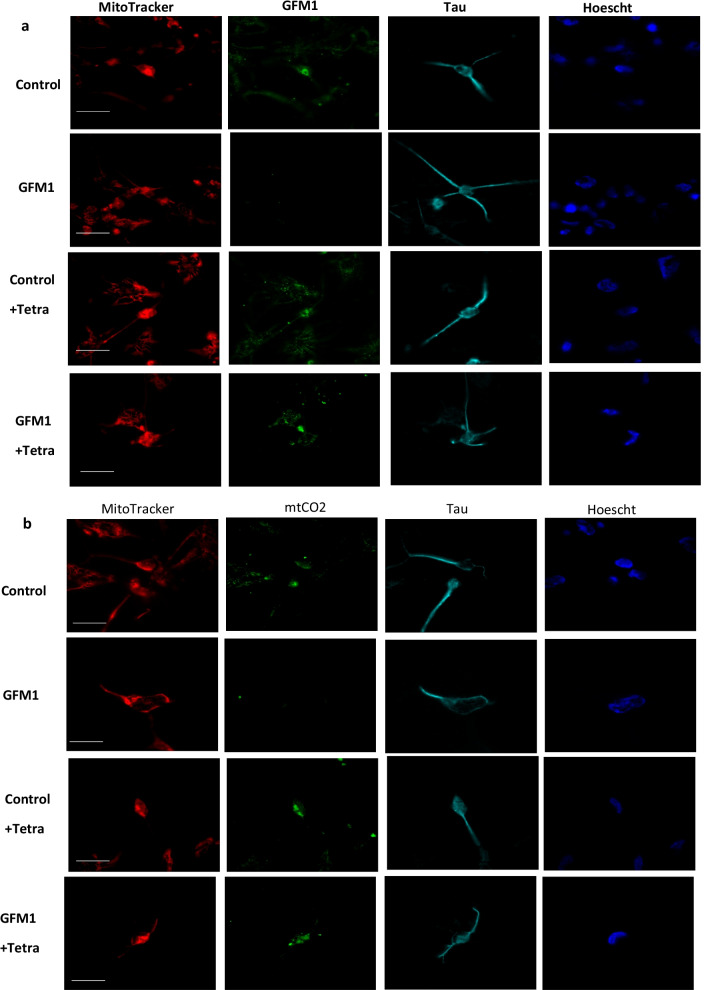
Mitochondrial protein expression and network in control and mutant *GFM1* induced neurons (iNS). Image analysis of Mitotracker DeepRed FM and EF-GF1 (**a**)/mtCO2 (**b**) in control and mutant *GFM1* iNS. Control and Mutant *GFM1* iNS were incubated with Mitotracker DeepRed FM 100 nM for 45 min, then they were fixed and immunostained with anti-mtCO2 (mitochondrial encoded complex IV subunit) or EF-GF1 (mutated protein) and Tau (neuronal marker). Then, they were examined by fluorescence microscopy. Nuclei were revealed by Hoescht 1 μg/ml staining. iNs were treated with 1 µM tetracycline for 7 days. Scale bar = 15 μm. Fluorescence intensity quantification versus cell area shown in Additional file 1: Figure S9

We then evaluated the efficacy of tetracycline treatment in mutant *GFM1* iNs. In this case, tetracycline concentration was reduced to 1 μM, since high doses could affect the viability of these cells [60]. Such concentration was selected according to our previous experiments in fibroblasts, where it was the lowest concentration promoting cell survival (Additional file 1: Figure S2). EF-GF1 (Fig. 8a and Additional file 1: Figure S9a) and mt-CO2 (Fig. 8b and Additional file 1: Figure S9b) protein expression levels were assessed by confocal microscopy. Additionally, mitochondrial network integrity was assessed by MitoTracker Deep Red FM staining. In mutant *GFM1* iNs, EF-GF1 and mt-CO2 were almost absent compared to controls. Interestingly, tetracycline treatment partially reverted the disease phenotype on mutant *GFM1* iNs as seen in fibroblasts.

### Tetracycline treatment highly modifies the transcriptome

Aiming to assess the effect of tetracycline on gene expression we decided to perform an RNA-Seq experiment on fibroblasts. Amidst the 60.675 expressed genes that were detected, 11.080 showed differential expression in control and mutant *GFM1* cells. The relative expression of all the differentially expressed genes between treated and non-treated controls (a) and between treated or untreated mutant *GFM1* cells (b) is depicted in Additional file 1: Figure S10. Moreover, control cells are significantly more sensitive to tetracycline treatment (Additional file 1: Figure S10c) than mutant *GFM1* cells (Additional file 1: Figure S10d) as evidenced by more prominent changes in their gene expression profile. Since the human UPR^mt^ activation pathway remains obscure, no databases like the Kyoto Encyclopedia of Genes and Genomes (KEGG) are available to study the proteins involved in it. For this reason, biological process ontology (BP) (Additional file 1: Figure S10e) and KEGG pathway ontology (Additional file 1: Figure S10f) were used to assess which of the differentially expressed genes might be related with UPR^mt^ activation. BP analysis demonstrated that mutant cells most prominently express genes related to chaperones, cGMP and cAMP production while KEGG analysis revealed that they present a higher expression of genes involved in pathways linked to calcium signalling, cGMP and Phosphoinositide 3-kinases (PI3K)-Protein kinase B (AKT) cascades and thiamine or retinol, which are essential cofactors for mitochondrial proteins [61]. Even though no direct connection between these pathways and UPR^mt^ has been reported so far, it is known that they are fundamental for the maintenance of mitochondrial function and homeostasis [62–64].

Next, UPR^mt^-related gene expression levels were examined including *eif2alpha*, *ATF4*, *ATF5*, *CHOP*, *HSP60*, *mtHSP70*, *SIRT3*, *PGC1α*, *Nrf2 *and *TFAM* (Additional file 1: Figure S11a). Only *HSP60*, *mtHSP70* and *TFAM* were significatively decreased in *GFM1* versus control cells. In contrast, *ATF5*, *CHOP* and *SIRT3* were upregulated in *GFM1* versus control cells, suggesting the activation of a compensatory mechanism. After tetracycline treatment, expression levels were markedly increased both in control and *GFM1* cells.

## Discussion

The therapeutic use of antibiotics has always been controversial due to their possible deleterious impact on mitochondrial function [65], given that they could even aggravate or trigger the appearance of hidden severe mitochondrial pathologies [66, 67] or mitochondrial-related diseases [68, 69]. Moreover, the extensive application of antibiotics would greatly contribute to the selection of resistant bacterial strands [70], which could represent a serious public health problem. Tetracyclines possess many properties that make them ideal antibiotic drugs, including activity against Gram-positive and -negative pathogens, proven clinical safety, acceptable tolerability, and the availability of intravenous and oral therapeutic formulations [71]. Tetracyclines’ antibiotic properties are based on their ability to bind bacterial ribosomes, where they interact with a highly conserved 16S ribosomal RNA (rRNA) target located in the 30S ribosomal subunit, therefore arresting translation by sterically interfering with the docking of aminoacyl-transfer RNA (tRNA) during elongation [30]. On top of being potent antibiotics, tetracyclines present antioxidant activity [72], metalloproteases inhibitory capacity [73], antiapoptotic features [74], anti-inflammatory characteristics [75] and mitochondrial enhancement potential [32]. For this reason, there is an increasing amount of studies supporting their therapeutic use for disorders other to bacterial infections such as cancer [76–78], renal alterations [79], aging [41, 80] and neurodegenerative diseases [81]. Even though several authors warn about the possible damage that their use might infringe on patients [82, 83], it must be pointed out that just like for many other commercial drugs: *“sola dosis facit venenum”.* In summary, tetracyclines’ toxicity and side-effects depend largely on the administered dose, being low concentrations of the drug presumably capable of inducing an hormetic effect on cells.

Mitohormesis is defined as a biological response at which the induction of minor mitochondrial stress leads to an increment in health and viability of a cell, tissue, or organism [27]. The response to mitochondrial stress triggered by potentially harmful stimuli occurs as a result of the coordinated interplay between mitochondria and the nucleus. This molecular “dialogue” is possible thanks to either: the release of mitochondrial ROS [84], mitoquines [85] or the activation of pathways such as UPR^mt^ [37], being most likely all these mechanisms involved to different extent. It has been demonstrated that the mitohormetic effect prolongs lifespan of several animal models, from worms to mammals [27]. To date, the pathways responsible for this phenomenon in humans are not fully understood but several studies suggest using it as a therapeutic tool for various diseases [86]. Our results show that tetracycline could induce an hormetic response in mutant *GFM1* cells in the same manner. This particular drug was selected among other therapeutic candidates because (1) it is known to activate UPR^mt^ in animal models [87]; (2) it’s a highly studied approved drug; (3) its activity on mitochondria has a similar impact to that of *GFM1* mutation. Indeed, the *GFM1* gene has a high phylogenetic resemblance to the bacterial tetracycline-resistance mechanisms TET(O) and TET(M) [71], pointing towards the fact that the response to tetracycline- induced stress must be very similar to that of *GFM1 *mutation-related damage. Thereafter, tetracycline application would boost the activation of the mitochondrial homeostasis machinery and hence, indirectly palliate pre-existing pathological alterations. In fact, treatment with very low doses of mitochondrial toxins like rotenone or paraquat have been reported to have a beneficial effect on mitochondrial function [88, 89]. This observation suggests that mitohormesis could explain the efficacy of numerous molecules having a favourable impact on the performance of our organism [90, 91].

Mutant *GFM1* cells present all the necessary features to be considered a good mitochondrial disease model: (1) *GFM1* mutation triggers an easily quantifiable decrease in the amount of mitochondrial proteins as well as compromises mitochondrial function, (2) mutant cells grow well in glucose-rich media, (3) a 3-day screening in galactose medium is sufficient to know whether a particular treatment is efficacious or not, (4) high reproducibility of assays irrespective of time differences. Taking into account these advantages, we started a pharmacological screening on mutant *GFM1* cells. We initially treated mutant cells with cofactors, antioxidants or mitochondrial function enhancers. However, none of them succeeded on promoting the survival of these cells in galactose medium. These results suggest that such compounds, even though being mildly beneficial for mitochondria, are not enough to trigger a cellular compensatory response to the damage infringed by *GFM1* mutation. The idea of promoting cellular compensatory mechanisms is not novel in the field. It was already proposed in a study that aimed to activate mitophagy and autophagy to remove mitochondria with mutant DNA in mitochondrial heteroplasmy diseases [92]. Thanks to mTOR inhibition with rapamycin researchers were able to increase the selective pressure over mitochondria and, as a consequence, to decrease the mutant mtDNA population. This led to a significant improvement in ATP production, mitochondrial activity, muscle endurance and lifespan of animal models and cell cultures [93, 94]. Rapamycin efficacy lies on its ability to elicit a moderate mitochondrial stress that promotes mitohormesis and thus, a better cellular function. This is presumably also the mechanism explaining tetracyclines’ therapeutic potential. Our results suggest that tetracycline triggers mitohormesis through the activation of UPR^mt^, which has been thoroughly studied in animal models [34, 87] but is not fully understood in humans yet. It is known that the proteins ATF4, ATF5 and CHOP [44] are an important regulatory axis in this pathway but their specific function and link to other pathways remains elusive. Several studies point out the relevance of ATF5 for cell survival [95] while others claim it is a pro-apoptotic protein [96]. Such discrepancy might be explained by the possibility of ATF5 having a dual function that depends on the level of mitochondrial damage, promoting protein homeostasis and mitophagy upon mild damage and apoptosis when it is severe or long-lasting. In this respect, *ATF5* silencing enabled mutant *GFM1 *cells’ survival in galactose and triggered an increase in the levels of other UPR^mt^ -related proteins such as ATF4. This observation supports the relationship between ATF5 and cell survival [97]. The ability of *ATF5* silencing to enhance cell survival was also observed in a previous study investigating the effect of proteasomal inhibition [96].

The results of this study prove that tetracycline rises the levels of UPR^mt^—related proteins in treated cells and promotes the activation of pathways involving cAMP and cGMP (Additional file 1: Figure S9e), which might be implicated in mitochondrial compensatory mechanisms comprising sirtuins and chaperones’ activity. Mitochondrial cAMP signalling is an indispensable part of the cytoplasm-mitochondrion crosstalk, which maintains mitochondrial homeostasis, regulates mitochondrial dynamics, and modulates cellular stress responses and other signalling cascades [63]. The cytosolic cAMP- Protein kinase A (PKA) pathway can also activate the nuclear CREBs, also known as ATFs, which are involved in the UPR^mt^ core, and the downstream transcription factors (PGC-1α, NRF), which in turn promote mitochondrial biogenesis and the transcription of *TFAM* [98, 99], which are also increased in *GFM1* cells after tetracycline treatment (Fig. 6). CREBs can be found inside mitochondria, binding to the CREBs on the mtDNA D-loop, and directly regulating mtDNA gene expression [100]. The translocation of CREBs into mitochondria may be facilitated by chaperones such as mtHSP70 [101] or by a process that depends on both, membrane potential and a translocase complex of the outer membrane (TOM) [100]. Both nuclear and mitochondrial CREB pathways promote neuronal survival in the brain [102], which is consistent with mitochondrial function enhancement. For these reasons, tetracyclines have been proposed as a treatment for neurodegenerative disorders [103] such as Parkinson’s disease [81]. On the other hand, cGMP signalling has been linked to mitochondrial biogenesis [104] and CREBs activation [62]. A study using a myotubular cell model showed that cGMP increased mitochondrial density while lowering ROS production [105]. Moreover, this molecule promoted the expression of genes participating in mitochondrial biogenesis, function and maintenance such as *PGC1α*, *Nrf1*, *ATP synthase*, and *COX4*, as well as that of genes contributing to ROS reduction, including Mitochondrial uncoupling protein 3 (*UCP3*) and Superoxide dismutase 2 (*SOD2*), and their up-stream regulator, peroxisome proliferator activated receptors (*PPAR*) delta and *CREB-1* [106]. In fact, treatments that augment cGMP-dependent signalling cascades have been proposed to attenuate mitochondrial dysfunction [62, 107–109]. Overall, we could prove the pleiotropic effect of tetracyclines on several mitochondrial hormesis-related pathways in which CREB/ATF proteins are involved.

According to our results *GFM1* mutations may yield an aberrant protein that is detected and prematurely degraded by mitochondrial quality control mechanisms [110]. As a consequence, mutant cells present a severe mitochondrial disease phenotype. However, upon tetracycline treatment and the subsequent activation of UPR^mt^, the increased number of chaperones and mitochondrial auxiliary proteins may promote the stability of a fraction of EF-G1 proteins which would carry out their function to some extent. The slight increase in the protein’s levels would be sufficient to boost mitochondrial function and cell survival in galactose. The activation of UPR^mt^ and its implication in mitochondrial protein quality control has already been described as a vital factor that if impaired, can lead to diseases such as Parkinson’s and Huntington’s disease [111]. On the other hand, some studies highlight the risks associated to a continuous activation of UPR^mt^ [112–114], nonetheless, these works focused exclusively on a possible modification of the function of *ATFS1*, the worm analogue for *ATF5* and *ATF4*, but do not assess the whole pathway.

It must also be pointed out that the continuous activation of compensatory pathways in healthy cells could have undesirable side effects, since they would alter mitochondrial homeostasis. While our findings support an alternative therapeutic use for antibiotics, it should be kept in mind that tetracycline, like other anti-microbial drugs may not be optimal for long-term treatments since it could infringe hepatic and renal damage and could give rise to resistant bacterial strands. If antibiotic treatments were to be used in the clinic, it might be advisable to apply them at sub-antibiotic doses. The sub-antibiotic dose of tetracycline, that below which it does not exert antibiotic activity has been established between 1-5 μM depending on the bacterial type [115, 116]. Our experiments were carried out with a tetracycline dose of 10 μM but a positive effect could also be observed at doses as low as 100 nM. Chronic tetracycline treatment has already been considered for clinical application due to tetracycline’s anti-inflammatory properties and its MMP inhibitory activity [117–119]. Overall, promoting the activation of compensatory pathways such as the UPR^mt^ could be beneficial for mitochondrial diseases patients, hence, further research should be conducted on the field for the discovery of non-antibiotic UPR^mt^ enhancing molecules with less side effects.

## Conclusion

Currently, there is no effective treatment for most mitochondrial diseases, only palliative therapies based on antioxidants and cofactors are available. The ideal solution for these diseases would undoubtedly be gene therapy [120], however, this technology is not likely to be applied for the treatment of complex human diseases in the near future. In this study we identified a mitochondrial disease cellular model unable to survive in galactose medium. This feature represented a significant advantage since it allowed for fast and precise drug screenings. The most promising therapeutic compound identified was tetracycline. Such antibiotic seemingly activates UPR^mt^, a mitochondrial homeostasis compensatory pathway, and by doing so reduces the pathogenicity of *GFM1* mutation and enhances mitochondrial function in patients’ fibroblasts. Our results support a new therapeutic approach against mitochondrial diseases going far beyond the traditional supplementation of active compounds. The recent discoveries on mitohormesis and the adaptative capacity of the mitochondrial machinery will surely be the starting point for future research on the field, as well as of better, more efficacious treatments.

## Materials and methods

### Reagents

The following antibodies were purchased from Abcam (Cambridge, UK): GFM1 (ab173529), mt-ND1 (ab181848), UQCRC1 (ab110252), mt-CO2 (ab79393), VDAC (ab14734), actin (ab8226), ATP5A1 (ab14748), COX4 (ab14744), ATF5 (ab184923), SIRT3 (ab217319), ATF4 (ab184909), CHOP (ab11419), Nrf1 (ab175932), TSFM (ab173528).

mtHSP70 antibody (MA3-028), HSP60 antibody (MA3-012), Tau antibody (MN1000), MitoTracker Deep Red FM (M22426), Donkey anti-Rabbit IgG (H+L) Highly Cross-Adsorbed Secondary Antibody, Alexa Fluor 555 (A-31572) and Donkey anti-Mouse IgG (H+L) Highly Cross-Adsorbed Secondary Antibody, Alexa Fluor 488 (A-21202) were purchased from Thermo Fisher (Waltham, MA, USA).

eif2α (5324) and P-eif2α (9721) antibodies were purchased from Cell Signaling (Danvers, MA, USA).

ATF4 shRNA (sc-35112-V), ATF5 shRNA (sc-43580-V), scramble shRNA (sc-108080), galactose (sc-202564), paraformaldehyde (sc-253236B), rotenone (sc-203242), oligomycin (sc-203342), antimycin A (sc-202467A), FCCP (sc-203578), DAPI (sc-3598) and HEPES (sc-29097) were purchased from Santa Cruz Biotechnology (Santa Cruz, CA, USA).

Tetracycline (87128-25G), minocycline (M9511-25MG), doxycycline (D3447-500MG), saponin (S7900-25G), valproic acid (P4543-10G), LDN-1931189(SML0559-5MG), Db-cAMP (D0260-100MG), CHIR99021 (SML1046-5MG), Goat Anti-Rabbit IgG H&L (HRP) (401353-2 ml), Goat Anti-Mouse IgG, H&L Chain Specific Peroxidase Conjugate (401253-2 ml) and donkey serum (D9663) were purchased from Merck (Darmstadt, Germany).

SB431542 (1614/10), Noggin (6057-NG-100), LM-22A4(4607/10), GDNF (212-GD-010) and NT3 (267-N3-025) were purchased from R&D systems (Minneapolis, MN, USA).

PBS (Phosphate Buffer Saline, 102309) 10 × was purchased from Intron Biotechnology (Seongnam, South Korea) and then diluted to 1 × PBS pH 7.4. BSA (Bovine Serum Albumin, A6588.0100) was purchased from Applichem (Darmstadt, Germany).

### Ethical statements

Approval of the ethical committee of the Hospital Universitario Virgen Macarena y Virgen de Rocío de Sevilla (Spain) was obtained, according to the principles of the Declaration of Helsinki as well as the International Conferences on Harmonization and Good Clinical Practice Guidelines.

### Fibroblast cultures

Cultured fibroblasts were derived from a skin biopsy of patients (GFM1, MAGF1, GAGF1 and TSFM) with the following mutations:

- GFM1: heterozygous mutation c.1404delA, p. (Gly469Valfs*84), in exon 12 (NM_024996.5, OMIM 606639) and c.2011C > T, p. (Arg671Cys), in exon 16 (NM_024996.5, OMIM 606639) of the *GFM1* gene.
- MAGF1 and -GAGF1 are from brothers bearing: heterozygous mutation c.179C > G, p. (Thr60Ser), in exon 2 (NM_001308164.1, OMIM 606639) and c.2068C > T, p. (Arg690Cys), in exon 17 (NM_001308164.1, OMIM 60639) of the *GFM1* gene.
- TSFM: homozygous mutation c.719G > C, p. (Cys240Ser), in exon 6 (NM_005726.5, OMIM 604723) in the *TSFM* gene. *TSFM* cells were a generous donation from Julio Montoya (Zaragoza University).

Control fibroblasts were human skin primary fibroblasts from healthy volunteer donors. Samples from patients and controls were obtained according to the Helsinki Declarations of 1964, as revised in 2001. Fibroblasts from patients and controls were cultured at 37 °C in DMEM (Dulbecco’s Modified Eagle Medium; Fisher Scientific, Waltham, MA, USA, 10524684) containing 4.5 g·L − 1 glucose, L-glutamine, and pyruvate supplemented with 1% antibiotic Pen-Strep solution (Thermo Fisher, Waltham, MA, USA, 11548876) and 20% Fetal Bovine Serum (FBS) (Thermo Fisher, Waltham, MA, USA, 10270-106). All the experiments were performed with fibroblasts cell cultures with a passage number < 8.

### *ATF4* and *ATF5* silencing

Cells were seeded in 12-wells plates and grown in DMEM medium with 4.5 g L^−1^ glucose, 10% fetal bovine serum and 1% antibiotics (Optimal medium). After reaching a 50% confluency they were washed once with PBS before being cultured in optimal medium supplemented with 10 μg/ml of Polybrene (Santa Cruz Biotechnology, Santa Cruz, CA, USA, sc-134220). shRNA Lentiviral particles (Santa Cruz Biotechnologies, Santa Cruz, CA, USA) were subsequently added to the culture (shControl/shATF4/shATF5) and incubated overnight at 37 °C, 5% CO_2_. The following morning cells were washed with PBS once and then kept in optimal medium overnight at 37 °C, 5% CO_2_. On the following day the content of each well was split in 3 different T25 Flasks and subjected to further incubation for 48 h in optimal medium (37 °C, 5%CO_2_). To select transfected and stable clones, the medium was supplemented with 2 μg/ml puromycin (Santa Cruz Biotechnology, Santa Cruz, CA, USA, sc-108071). Both Puromycin and culture medium were refreshed every 3 days.

### Galactose screening

When galactose is the only carbon source in the culture medium, cells either metabolize it via glycolysis or OXPHOS [121]. Whereas glucose metabolism via glycolysis yields 2 net ATP, the glycolytic breakdown of galactose yields no net ATP, forcing cells to rely on oxidative phosphorylation (OXPHOS) for energy supply [122]. For this reason, cells whose mitochondrial function is compromised will rarely grow or survive in a glucose-free galactose medium, unlike healthy cells. Thus, culturing cells in a galactose medium is a powerful tool to study mitochondrial dysfunction and carry out drug screening experiments.

Galactose medium was prepared with DMEM no glucose (Fisher Scientific, Waltham, MA, USA, A1443001) supplemented with 20 mM galactose, 15 mM HEPES, 1% Pen-Strep solution and 10% FBS. Cells were seeded in 24-well plates in optimal medium. After 24 h, cells were treated for 72 h with different drugs. Then medium was removed and cells were washed with PBS prior to the addition of the galactose medium (T0). Then, the treatments were re-applied in the same concentration.

Cell viability was assessed by live cell imaging counting and trypan blue 0.2% staining. Cell counting and representative images were acquired using the BioTek™ Cytation™ 1 Cell Imaging Multi-Mode Reader (Biotek, Winooski, VT, USA).

### Immunofluorescence microscopy

Treated and untreated fibroblasts were grown on 1 mm width glass coverslips for 72 h in normal growth medium with/ without the addition of 10 µM tetracycline. Three replicates per condition were performed. Cells were stained with 100 nM MitoTracker DeepRed FM 45 min before fixation. Afterwards, they were washed twice with PBS, fixed in 3.8% paraformaldehyde for 15 min at room temperature, incubated in blocking buffer (BSA 1% in PBS) and permeabilized with 0.1% saponin in blocking buffer for 1 h. In the meantime, primary antibodies were diluted 1:100 in antibody buffer (BSA 0.5% plus saponin 0.1% in PBS). Fibroblasts were incubated overnight at 4 °C with the antibodies and subsequently washed twice with PBS. Secondary antibodies were similarly diluted 1:400 antibody buffer, but their incubation time on cells was reduced to 2 h at room temperature. Coverslips were then washed twice with PBS, incubated for 5 min with PBS containing DAPI 1 µg/ml and washed again with PBS. Next, they were mounted on microscope slides using Vectashield Mounting Medium (Vector Laboratories, Burlingame, CA, USA, H1000).

Neurons were grown on μ-SLIDE 4-well ibitreat plates (Ibidi, Gräfelfing, Germany, 80426) and stained with 100 nM MitoTracker Deep Red FM 45 min before fixation. One μ-SLIDE 4-well ibitreat plate per condition was used. Cells were washed with PBS, fixed in 4% paraformaldehyde for 10 min at room temperature, and permeabilized with 0.1% Triton X-100 for 10 min. Then, blocking buffer consisting on PBS 5% donkey serum was added for 1 h. Primary antibodies were diluted 1:100 in PBS 5% donkey serum and incubated on the cells overnight at 4ºC. The following morning neurons were washed twice with PBS prior to the addition of the secondary antibodies. These were diluted 1:300 in PBS 5% donkey serum and incubated for 2 h at room temperature. Finally, cells were washed twice with PBS, incubated for 15 min with PBS containing DAPI dilution 1 µg/ml and washed with PBS.

50 cells per condition were specifically selected. Samples were analyzed using an upright fluorescence microscope (Leica DMRE, Leica Microsystems GmbH, Wetzlar, Germany). Images were taken using a DeltaVision system (Applied Precision; Issaquah, WA, USA) with an Olympus IX-71 microscope using a 100 × objective. Images were analysed using the softWoRx and ImageJ software.

### Protein synthesis

Treated and untreated fibroblasts were grown on 1 mm width glass coverslips for 72 h in optimal growth medium with or without the addition of 10 µM tetracycline. Cells were stained with 100 nM MitoTracker DeepRed FM 45 min before fixation. To check mitochondrial and cytoplasmatic protein synthesis, cells were treated with chloramphenicol 150 µg/ml for 50 min and/or cycloheximide 50 µg/ml for 20 min. Then, we followed the protocol provided with the Click-iT® HPG 488 Alexa Fluor® Protein Synthesis Assay Kit (Life technologies, Carlsbad, CA, USA, C10428). Briefly, cells were incubated with the alkyne-containing non-canonical amino acid L-homopropargylglycine (HPG). Under these conditions, HPG is specifically incorporated into mitochondrial translation products instead of methionine and can be visualized by a subsequent copper-catalyzed cycloaddition reaction (click) to azide-containing fluorescent dyes [123].

20 cells per condition were specifically selected. Samples were analysed using an upright fluorescence microscope (Leica DMRE, Leica Microsystems GmbH, Wetzlar, Germany). Images were taken using a DeltaVision system (Applied Precision; Issaquah, WA, USA) with an Olympus IX-71 microscope using a 100 × objective. Images were analyzed using the ImageJ software.

### Direct reprogramming

Neurons were generated from mutant *GFM1* and control fibroblasts by direct neuronal reprogramming as previously described by Drouin-Ouellet et al. [124].

Controls and mutant *GFM1* patient-derived fibroblasts were plated on μ-Slide 4-Well Ibidi plates and cultured in DMEM + Glutamax [61965059] with 1% Pen-Strep solution and 10% FBS. The day after, dermal fibroblasts were transduced with one-single lentiviral vector containing neural lineage-specific transcription factors (*ASCL1* and *BRN2*) and two shRNA against the REST complex, which were generated as previously described with a non-regulated ubiquitous phosphoglycerate kinase (PGK) promoter [125]. The plasmid was a gift from Dr. Malin Parmar (Developmental and Regenerative Neurobiology, Lund University, Sweden). Transduction was performed at a multiplicity of infection (MOI) of 30. On the following day cell culture medium was switched to fresh DMEM and after 48 h to neural differentiation medium (NDiff227; Takara-Clontech, Kusatsu, Japan, Y40002) supplemented with neural growth factors and small molecules at the following concentrations: LM-22A4 (2 μM), GDNF (2 ng/mL), NT3 (10 ng/mL), dibutyryl cyclic AMP (db-cAMP, 0.5 mM), CHIR99021 (2 μM), SB-431542 (10 μM), noggin (50 ng/mL), LDN-193189 (0.5 M) and valproic acid (VPA, 1 mM). Half of the neuronal differentiation medium was refreshed every 2–3 days. Eighteen days post-infection (DPI), the medium was replaced by neuronal medium supplemented with only growth factors until the end of the cellular conversion. At day 21, cells were treated with 1 μM tetracycline and the medium was changed every 2–3 days for 10 more days. Neuronal cells were identified by the expression of Tau protein, using the anti-TAU clone HT7 antibody. Nuclei were stained with DAPI (Invitrogen/Molecular Probes, Eugene, OR, USA, D1306). DAPI+/Tau+ cells were considered iNs. Conversion efficiency was calculated as the number of Tau+ cells over the total number of fibroblasts seeded for conversion. Neuronal purity was calculated as the number of Tau+ cells over the total cells in the plate after reprogramming.

### Immunoblotting

Western blotting was performed using standard methods. After transferring the proteins to nitrocellulose membranes (BIORAD, Hercules, CA, USA, #1620115), the membranes were incubated with primary antibodies, which were diluted 1:1000 in BSA 5% overnight, washed twice with TTBS and incubated with the corresponding secondary antibody for 1 h at 4 °C. Secondary antibodies were diluted 1:2500 in BSA 5%. Multiple blots were run and several proteins of interest were serially detected. Every membrane was checked for protein loading using Ponceau staining and actin protein levels. Stripping was not used. If possible, membranes were re-probed with different antibodies. This is when the molecular weight of the new protein of interest did not interfere with that of the previous one. Moreover, if the proteins were sufficiently separated from one another during gel electrophoresis, membranes were cut and each respective piece was used to detect a different target protein.

### Bioenergetics

Mitochondrial respiratory function of control and mutant *GFM1* fibroblasts was measured using a mito-stress test assay by XF24 extracellular flux analyzer (Seahorse Bioscience, Billerica, MA, USA, 102340-100) according to the manufacturer's instructions. Cells were seeded at a density of 1.5 × 10^4^ cells/well with 500 µL growth medium (DMEM medium containing 10% FBS and 4,5 g/l glucose) in XF24 cell culture plates and incubated for 24 h at 37 °C, 5% CO_2_. Subsequently, growth medium was removed from the wells, leaving on them only 50 µL medium. Then, cells were washed twice with 500 µL of pre-warmed assay medium XF base medium [102353-100] supplemented with 10 mM glucose [103577-100], 1 mM glutamine [103579-100] and 1 mM sodium pyruvate [103578-100]; pH 7.4) and eventually 450 µL of assay medium (500 µL final) were added. Cells were incubated at 37 °C without CO_2_ for 1 h to allow pre-equilibrating with the assay medium. Mitochondrial functionality was evaluated by sequential injection of four compounds affecting bioenergetics. The final concentrations of the injected reagents were: 1 µM oligomycin, 2 µM FCCP, 1 and 2.5 µM rotenone/antimycin A. The best concentration of each inhibitor and uncoupler, as well as the optimal cells seeding density were determined in preliminary analyses. A minimum of five wells per treatment were used in any given experiment. This assay allowed for an estimation of basal respiration, maximal respiration and spare respiratory capacity. Normalization was performed by cell counting after the assay using DAPI staining and using the BioTek™ Cytation™ 1 Cell Imaging Multi-Mode Reader.

#### Mitochondrial complexes activity

Activity of complex I and complex IV was assessed according to the protocol of the Complex I (ab109720)/Complex IV (ab109876) Enzyme Activity Dipstick Assay Kit (Abcam, Cambridge, UK) starting from a cellular pellet. In this technique the proteins from cellular lysates migrate through a nitrocellulose membrane. Then, Complex I is immunocaptured (i.e. immuno-precipitated in active form) on the dipstick. Then, the dipstick is immersed in Complex I activity buffer solution containing NADH as a substrate and nitrotetrazolium blue (NBT) as the electron acceptor. Immunocaptured complex I oxidizes NADH and the resulting H+ reduces NBT to form a blue-purple precipitate at the Complex I antibody line on the dipstick. The signal intensity of this precipitate corresponds to the level of Complex I enzyme activity in the sample. 30 μg of protein were used for each assay, according to the protocol range. Three replicates were performed for each respiratory complex assay.

Images of the dipsticks were acquired with a Molecular Imager ChemiDoc XRS+ System (BIORAD, Hercules, CA, USA) and quantified with the Image Lab software.

#### RNAseq

From cellular pellets, RNA was extracted and purified using RNeasy Mini Kit (QIAGEN, Hilden, Germany). Also, DNase digestion was performed with the RNase-Free DNase Set (QIAGEN, Hilden, Germany). RNAseq was performed by Microomics Systems S.L. (Barcelona, Spain). Enrichment Score was calculated using Merico et al. 2010 methods [126].

#### Statistical analysis

We used non-parametric statistics, which do not take into consideration distributional assumptions, given the low reliability of normality testing for small sample sizes like those used in this work [127]. To compare parameters between groups, variables were evaluated using the Wilcoxon match-paired signed rank test, the Friedman Test or a 2-way ANOVA Test. All results were expressed as mean ± standard deviation (SD) of 3 independent experiments and a p-value < 0.05 was considered as statistically significant.

## Supplementary Information

**Additional file 1.**Supplementary figures.

## Data Availability

Data and material are available under request.

## Abbreviations

ASCL1: Achaete-Scute Family BHLH Transcription Factor 1
ATF4: Activating Transcription Factor 4
ATF5: Activating Transcription Factor 5
ATFS-1: Activating Transcription Factor associated with Stress 1
ATP: Adenosine triphosphate
ATP5A1: ATP synthase subunit alpha
BRN2: Brain-Specific Homeobox/POU Domain Protein 2
BSA: Bovine Serum Albumin
CHOP: C/EBP homologous protein
COX4: Cytochrome c oxidase subunit 4
CREB-2: Cyclic AMP-Responsive Element Binding Protein 2
DMEM: Dulbecco’s modified Eagle medium
EF-G1: Elongation Factor G 1
eif2α: Eukaryotic Initiation Factor 2
FBS: Fetal Bovine Serum
FCCP: Trifluoromethoxy carbonyl cyanide phenylhydrazone
FUNCAT: Fluorescent non-canonical amino acid tagging
GDNF: Glial derivate neurotrophic factor
GFM1: G elongation factor mitochondrial 1
HPG: L-homopropargylglycine
HSP60: Heat Shock Protein 60
iNS: Induced Neurons
MMP: Metalloproteases
mt-CO2: Cytochrome C Oxidase 2
mtDNA: Mitochondrial DNA
mtHSP70: Mitochondrial Heat Shock Protein 70
mt-ND1: Mitochondrial NADH dehydrogenase 1
UPR^mt^: Mitochondrial Unfolded Protein Response
nDNA: Nuclear DNA
Nrf1: Nuclear Respiratory Factor 1
NT3: Neurotrophin 3
OCR: Oxygen Consumption Rate
OXPHOS: Oxidative Phosphorylation
PARP-1: Poly (ADP-ribose) polymerase-1
PBS: Phosphate Saline Buffer
P-eif2α: Phospho-Eukaryotic Initiation Factor 2
Pen-Strep: Penicillin Streptomycin
PGC1α: Peroxisome proliferator-activated receptor gamma coactivator 1-alpha
PGK: Phosphoglycerate kinase
shRNA: Short Hairpin RNA
SIRT3: Sirtuin 3
TFAM: Mitochondrial transcription factor A
TSFM: Ts Translation Elongation Factor, Mitochondrial
UQCRC1: Ubiquinol-Cytochrome C Reductase Core Protein 1
VDAC: Voltage Dependent Anion Channel
VPA: Valproic Acid
